# Embodied memory: unconscious smiling modulates emotional evaluation of episodic memories

**DOI:** 10.3389/fpsyg.2015.00650

**Published:** 2015-05-26

**Authors:** Mathieu Arminjon, Delphine Preissmann, Florian Chmetz, Andrea Duraku, François Ansermet, Pierre J. Magistretti

**Affiliations:** ^1^Agalma FoundationGeneva, Switzerland; ^2^Department of Psychiatry, Faculty of Medicine, University of GenevaGeneva, Switzerland; ^3^Institute of Psychology, University of LausanneLausanne, Switzerland; ^4^Centre for Psychiatric Neurosciences, Lausanne University Hospital CenterLausanne, Switzerland; ^5^Brain Mind Institute, École Polytechnique Fédérale de LausanneLausanne, Switzerland; ^6^Division of Biological and Environmental Sciences and Engineering, King Abdullah University of Science and TechnologyThuwal, Saudi Arabia

**Keywords:** somatic markers, memory, embodied cognition, facial feedback hypothesis, reconsolidation

## Abstract

Since Damasio introduced the somatic markers hypothesis in Damasio (1994), it has spread through the psychological community, where it is now commonly acknowledged that somatic states are a factor in producing the qualitative dimension of our experiences. Present actions are emotionally guided by those somatic states that were previously activated in similar experiences. In this model, somatic markers serve as a kind of embodied memory. Here, we test whether the manipulation of somatic markers can modulate the emotional evaluation of negative memories. Because facial feedback has been shown to be a powerful means of modifying emotional judgements, we used it to manipulate somatic markers. Participants first read a sad story in order to induce a negative emotional memory and then were asked to rate their emotions and memory about the text. Twenty-four hours later, the same participants were asked to assume a predetermined facial feedback (smiling) while reactivating their memory of the sad story. The participants were once again asked to fill in emotional and memory questionnaires about the text. Our results showed that participants who had smiled during memory reactivation later rated the text less negatively than control participants. However, the contraction of the zygomaticus muscles during memory reactivation did not have any impact on episodic memory scores. This suggests that manipulating somatic states modified emotional memory without affecting episodic memory. Thus, modulating memories through bodily states might pave the way to studying memory as an embodied function and help shape new kinds of psychotherapeutic interventions.

## Introduction

Folk conceptions often assume memory to be a ‘storehouse’ in which memories can be recollected exactly as they were encoded. Yet, this static model of memory has been disputed by scientists. More than 100 years ago, Müller and Pilzecker pointed out that newly acquired information can disrupt previously acquired memories (Lechner et al., 1999). In proposing the concept of memory consolidation, they emphasized memory’s dynamic nature. Memories are initially labile and need time to progressively consolidate, i.e., to become insensitive to interference. In recent decades, several studies in cognitive psychology and neuroscience have shown that even consolidated memories are not necessarily fixed and can be transformed. These observations gave rise to the reconsolidation theory, according to which even consolidated memories, when reactivated, return to a labile state and need reconsolidation processes to be re-stabilized (Nader, 2003; Dudai, 2006; Nader and Einarsson, 2010; Alberini, 2011). This theory fully accounts for the fact that memory is not a collection of realistic “photographs” of past events and sheds new light on why humans can form false memories (Loftus and Davis, 2006; Chan et al., 2009; Hardt et al., 2010). Instead of being a cognitive impairment, memory malleability appears to be critical for a fully adaptive memory, i.e., its ability to be updated according to new circumstances (Lee, 2009; Gisquet-Verrier and Riccio, 2012).

Evidence for the reconsolidation process is supported by observations of experimental amnesia provoked by various treatments given during memory reactivation. For instance, propranolol, a non-selective beta-blocker, has been shown to interfere with reconsolidation of negative emotional memory in healthy subjects and in patients with post-traumatic stress disorder (PTSD; Pitman et al., 2002; Kindt et al., 2009; Donovan, 2010; Debiec et al., 2011; Lonergan et al., 2012; Schwabe et al., 2012). Subjects that received propranolol before memory reactivation had reduced capacities to recall negative memories (Schwabe et al., 2012). These studies commonly assume that this effect is mainly due to propranolol’s interference with cellular reconsolidation mechanisms, in particular noradrenergic activity in the amygdala.

Yet, propranolol is also known to impact the sympathetic nervous system (i.e., decreased heart rate). This echoes the James-Lange peripheralist theory of emotions, to which Damasio has recently provided additional neurophysiological evidence (Damasio, 1996; Bechara et al., 2000a,b). According to Damasio’s somatic marker hypothesis, when an individual encounters new situations that evoke previous emotional experiences, or when emotional memories are recovered, the physiological states associated with the recalled event are automatically reactivated (Bechara et al., 2003). Such evidence fits well within the framework of “embodied cognition” which departs from mainstream cognitive science by positing that “cognition deeply depends on aspects of the agent’s body other than the brain.” (Shapiro, 2007; Wilson and Foglia, 2011). In line with the Damasio’s approach, we propose here that somatic markers constitute *embodied memories* and we ask to which extent this hypothesis offers a fresh perspective on the study of emotional memory malleability.

Indeed, in Damasio’s thesis, the memory of a frightening experience can be decomposed into two interdependent elements: (1) the episodic memory tied with the event and (2) the congruent somatic states (e.g., an elevated heart rate) associated with it. If we accept Damasio’s conceptual framework, one could ask whether peripheral somatic modifications are at least partly responsible for a decrease in intensity of posttraumatic symptoms – or more generally, fearful memories – when propranolol is administrated before memory reactivation. Yet, before testing this hypothesis, one ought to investigate whether manipulating somatic states during memory reactivation interferes with further emotional evaluations of the memory. In other words, will a negative memory be remembered as less negative for those whose bodily states were manipulated in order to mimic a pleasant emotion during memory reactivation?

Previous studies investigated the link between memory and somatic states. For instance, it has been shown that bodily states facilitate the accessibility of emotionally congruent memories: subjects recall more positive memories when smiling (Riskind, 1983) and remember more negative items if they sit in slumped positions during memory encoding (Michalak et al., 2014). These previous studies have explored to what extent the manipulation of somatic states impacts or biases accessibility to emotional memories. To our knowledge, however, no studies have investigated how somatic states manipulation can modulate the emotional evaluation of a memory.

To test the possible effect of somatic state on the evaluation of an emotional memory, we used facial feedback manipulation during memory reactivation. Several studies have demonstrated that manipulating facial feedback can affect emotional evaluation (Strack et al., 1988; Larsen et al., 1992; Alam et al., 2008; Davis et al., 2009). Importantly, these effects are observed even if subjects are unaware that they are producing a facial expression; i.e., smiling is induced by asking participants to hold a pen between teeth (Strack et al., 1988), frowning by looking at the sun (Marzoli et al., 2013), or reducing the distance between golf tees attached to the eyebrow (Larsen et al., 1992). For instance, Strack et al. (1988) showed that participants whose zygomaticus muscles were contracted by holding a pen between their teeth judged cartoons funnier than did non-smiling participants. More generally, it is worth mentioning that facial feedback has been shown to modulate sympathetic activations like heart rate and skin conductance (Soussignan, 2002; Lee et al., 2013) and to increase stress recovery, both at physiological and psychological levels (Kraft and Pressman, 2012). Since these studies highlight the immediate effect of facial feedback on emotional judgments, we hypothesized that facial feedback might also have an impact on further evaluation of emotional memories if manipulated during memory reactivation.

To test our hypothesis, we asked participants to read and rate their emotions about a negative text immediately after reading (day1) and 24 h after reading (day2). Unconscious smiling was induced during memory reactivation (day2) with a similar procedure as described in Strack et al. (1988), namely by holding a pen between the teeth (smiling), whereas control groups had no contraction of zygomaticus muscles. We predicted that smiling during reactivation should decrease the negative emotional evaluation of the text without affecting episodic memories.

## Materials and Methods

### Participants

Eighty-four healthy, French-speaking undergraduate students (41 males and 43 females, mean age 23.2 ± 0.54 SEM) were recruited at the University of Lausanne, Switzerland. All participants completed consent forms and received compensation for their participation (15 CHF). The local ethical committee approved the procedure.

We induced unconscious smiling in a manner similar to previous studies (Strack et al., 1988), by asking participants to hold a 12 mm diameter pen (Hedding 21) between their teeth (smiling group) or between their lips or with the non-dominant hand (control groups). Participants were randomly assigned to one of the three following experimental groups.

#### Unconscious Smile Group

**Teeth:** 31 participants (16 males and 15 females; mean age 22.4 ± 0.84 SEM). Participants were asked to hold the pen between their teeth without touching the lips. This manipulation produces a facial muscular activity comparable to a smile (contraction of the zygomaticus muscles), but the experimenter never mentioned the word “smiling” in the instruction.

#### Two Control Groups

**Lips**: 27 participants (12 males and 15 females; mean age 24.8 ± 1.30 SEM). Participants held the pen between their lips, thus holding the pen in their mouth in a position incompatible with a contraction of the zygomaticus muscles (smiling). **Non-dominant hand**: 26 participants (13 males and 13 females; mean age 22.4 ± 0.43 SEM). Participants were asked to perform the drawing task (see below) while holding the pen in the non-dominant hand. The Hand control group allowed us to assess whether holding a pen in the mouth had an effect independent of smiling.

### Procedure

**FIGURE 1:**
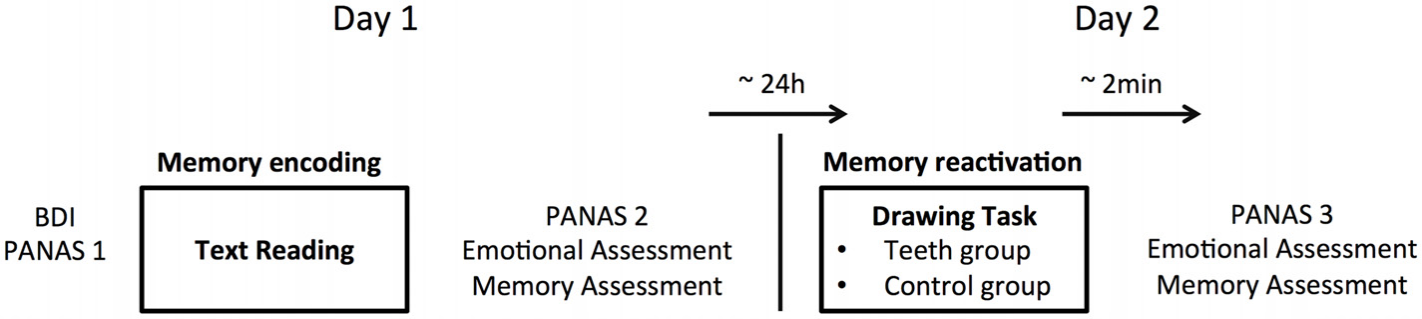
**Illustration of the experimental procedure. On day 1, participants filled in Beck’s Depression Inventory (BDI) and Positive and Negative Affect Schedule (PANAS) 1 questionnaires.** Then, participants read the text, performed an emotional evaluation and a memory evaluation of the text, and filled in PANAS 2. On day 2, participants performed a drawing task during which memory of the text was reactivated while facial feedback was manipulated. Two minutes after the drawing task, participants realized a second emotional evaluation and memory evaluation of the text and filled in PANAS 3.

Our experimental protocol took place across two sessions separated by approximately 24 h (**Figure 1**). All tests were conducted in the same quiet room.

#### Day 1

On the first day, the procedure of the experiment was described to the participants with a special emphasis on the drawing task and information about the way they were to hold the pen during the second day. We used a “multitasking study” cover story (Strack et al., 1988) to avoid awareness of smiling during the facial feedback manipulation. Participants were then asked to fill two questionnaires:

(1) The short form of the French version of the Beck’s Depression Inventory (BDI; Beck et al., 1961; Bourque and Beaudette, 1982). This thirteen items questionnaire assessed the level of depressive symptoms before experimental manipulation.
(2) The French version of the twenty-question Positive and Negative Affect Schedule (PANAS; Watson et al., 1988; Gaudreau et al., 2006) used to measure positive activation (PA) and negative activation (NA) at three points in time: before (PANAS 1) and after reading the text on the first day (PANAS 2) and after facial feedback manipulation on the second day (PANAS 3). The instructions for filling out PANAS were: “Answer according to how you feel at the present moment.”

After completing BDI and the first PANAS questionnaire, participants read a text excerpt from a casebook written by a pediatrician (Naouri, 2006). This excerpt of 1824 words relates the sad story of a woman who lived an unhappy childhood, contracts leukemia, and whose unfaithful husband had a child with her own sister. This text was chosen in order to induce a negative emotional memory in participants. Immediately after reading the text, participants once again completed the PANAS form. The comparison between the PANAS scores, before and after the reading, allowed us to measure the direct impact of the text on subject’s positive and NAs. As the text touched upon issues of unfaithfulness, marital status was considered in the analysis to ensure it had no effect on the emotional evaluation of the text (53 participants reported to be single and 31 reported to be in a relationship).

Next, participants were asked to fill out a questionnaire assessing their emotions regarding the text. Questions were rated on 10-point Likert scales. We used two questionnaires that were counterbalanced across the 2 days to avoid memory effects. Each item of the first questionnaire was related to an item of the second questionnaire with close face validity. The negative emotional evaluation toward the text was assessed with nine questions related to negative emotions like sadness or anger, e.g., “*This testimony makes me sad,*” “*This testimony makes me angry,*” “*The husband’s behavior is immoral*.”

Finally, the respondents completed a questionnaire assessing their memory of the text. We used two questionnaires with eight open questions that were designed to have a mild degree of difficulty (to avoid ceiling or floor effects). These questions were directly related to the text, for instance: “*How old is the first son of the couple when they come to consult the pediatrician for the first time?*” “*How many times a year does the woman see her family during her years in boarding school?*” As with the emotional assessment forms, the two questionnaires were counterbalanced between groups and sessions.

#### Day 2

On the second day, participants were asked to perform drawing tasks during which the manipulation of facial expression took place. We used drawing tasks similar to those described by Strack et al. (1988). The drawing tasks were displayed on paper sheets fixed to the wall at height of the head of the participants. The first task consisted of drawing a series of straight lines by connecting two numbered points. The second task involved drawing a line to connect 10 numbered points, randomly distributed across the paper sheet. The third task consisted of underlining vowels in a random series of 64 letters. The fourth task consisted of reading seven excerpts from the text and to indicate whether the excerpts were from the beginning, the middle or the end of the text. These text excerpts were presented in order to reactivate the memory of the text. Thus facial feedback was manipulated during memory reactivation and not during the emotional rating of the text. Participants were then asked to assess the difficulty of each task and the pleasantness of the whole drawing task. To be sure that facial feedback effects ceased between the two tasks, a 2-min delay was introduced between reactivation and rating.

After the drawing task, participants dropped the pen and filled out the PANAS 3, the emotional evaluation form and the memory evaluation questionnaire. At the end of the session, the experimenter asked participants if they had a hypothesis about the whole experiment (especially about the drawing task and the facial feedback procedure) to evaluate the efficiency of the cover story presented in the first session. None of the participants reported that the pen manipulation was meant to provoke a smile and no one guessed the real hypothesis of our study. Finally, participants were debriefed, paid and thanked for their participation.

## Results

All statistical analyses were conducted using SPSS software (IBM SPSS Statistics, Hong Kong, China; version 18.0).

We first performed separate analysis on control groups (lips and hand groups). As there was no difference between these two control groups, they were regrouped into a single *Control* group.

### Beck’s Depression Inventory and Positive and Negative Affect Schedule

There was no difference between groups in the level of depressive symptoms (BDI scores) at the beginning of the experiment (**Table 1**). A three-way ANOVA (group, sex, and marital status) showed no difference between groups [*F*(1,76) = 0.58, *p* = 0.44] nor sex [*F*(1,76) = 3.6, *p* = 0.06], but a significant effect of marital status, single people having higher scores than people in a relationship [*F*(1,76) = 4.35, *p* = 0.04].

**Table 1:** Mean scores of the Beck’s Depression Inventory (BDI), Positive and Negative Affect Schedule (PANAS) questionnaires, difficulty and unpleasantness assessment.

(A)	PANAS					
		Negative activation			Positive activation		
Groups	BDI	PANAS1	PANAS 2	PANAS 3	PANAS1	PANAS 2	PANAS 3
Controls	3.7 (±0.4)	17 (±0.7)	19.5 (±0.9)	15.5 (±0.7)	32.7 (±0.8)	29.6 (±0.9)	31.1 (±0.9)
Teeth	4.5 (±0.7)	18.6 (±1.4)	20 (±1.2)	16.4 (±1)	31.5 (±1.2)	30.6 (±1.4)	30.9 (±1.3)

**(B)**							
**Groups**	**Difficulty**		**Unpleasantness**

Teeth	5.4 (±0.3)		5.9 (±0.4)
Lips	5.5 (±0.3)		5.2 (+0.5)
Hand	3.6 (±0.3)		2.7 (±0.3)

**(A)** BDI and PANAS mean scores (±SEM). Both Control and Teeth groups showed an increase of negative activation (NA) and a decrease of positive activation (PA) between PANAS 1 and PANAS 2. There was no effect of facial feedback manipulation on PA and NA on PANAS 3. **(B)** Mean (±SEM) rating scores for the difficulty and unpleasantness of the task. Significant differences were found between Hand group and Teeth group as well as between Hand group and Lips group for difficulty and unpleasantness.

Both groups witnessed an increase of NA and a decrease of PA after the reading of the text (**Table 1**). This was confirmed by a two-way repeated measures ANOVA (group, sex as factors and NAs in PANAS 1 and 2 as repeated factor) showing a significant effect of the repeated measure [*F*(1,78) = 8.15, *p* = 0.006], no group effect [*F*(1,78) = 1.12, *p* = 0.29], no repeated measure^∗^groups interaction [*F*(1,78) = 0.75, *p* = 0.38] and a non-significant trend toward females reporting higher NAs [*F*(1,78) = 3.78, *p* = 0.055]. The decrease of PAs following the reading of the text was confirmed by a two-way repeated measures ANOVA (group, sex as factors and PAs in PANAS1 and 2 as repeated factor) showing a significant effect of the repeated measure [*F*(1,78) = 10.67, *p* = 0.002] but no group [*F*(1,78) = 0.009, *p* = 0.92] nor group*repeated measure interaction [*F*(1,78) = 3.41, *p* = 0.07] but a significant sex effect; female having lower PA than male participants [*F*(1,78) = 11.15, *p* = 0.001]. Finally, there was no significant effect of marital status on any PANAS questionnaires scores (*p* > 0.05) confirming that the text provoked similar emotional activations in participants independently of their marital status.

Separate ANOVAs on PANAS 3 (just after facial feedback manipulation) showed no effect of facial feedback manipulation on NA [*F*(1,79) = 1.21, *p* = 0.27] nor on PA [*F*(1,79) = 0.06, *p* = 0.8].

### Mean Rating of Task Difficulty and Unpleasantness

In contrast to the lack of difference in the emotional evaluation results between control participants holding the pen with the hand or with the mouth, a significant difference was found between these groups in the difficulty and unpleasantness ratings (**Table 1**).

Groups holding the pen in the mouth found the drawing more difficult to accomplish than the group holding the pen in the non-dominant hand (**Table 1**). This was confirmed by a two-way ANOVA (group, sex) which revealed a significant group effect [*F*(2,78) = 10.65, *p* < 0.0001] but no sex effect [*F*(1,78) = 0.7, *p* = 0.5]. *Post hoc* Bonferroni tests on the different groups showed that the hand group had a significantly lower difficulty score than both the teeth (*p* < 0.0001) and lips (*p* < 0.0001) groups, whereas no significant difference was found between teeth and lips groups (*p* = 1).

Groups who held the pen in their mouth (teeth and lips) also rated the drawing task to be more unpleasant (**Table 1**). This was confirmed by a two-way ANOVA (group, sex) showing a group effect [*F*(2,78) = 18.04, *p* < 0.0001] and no sex effect [*F*(1,78) = 0.38, *p* = 0.53]. *Post hoc* Bonferroni tests showed that the hand group rated the drawing task to be less unpleasant than both the teeth and the lips groups (*p* < 0.0001), whereas there was no significant difference between lips and teeth groups (*p* = 0.62).

### Emotional Evaluation and Memory Scores

**FIGURE 2:**
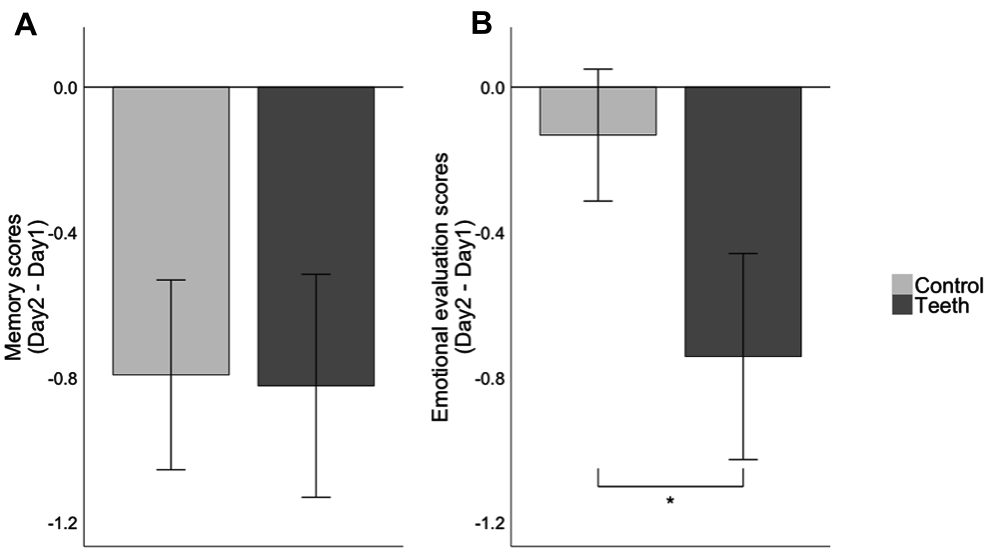
**Differential scores for memory and emotional evaluation.** Differential scores were calculated by subtracting day 2 scores to day 1 scores. Scores equal to 0 mean no difference across sessions and negative scores indicate a decrease from session 1 to session 2. **(A)** Differential scores for the memory of the text. **(B)** Differential scores of emotional evaluation of the text. Notes: error-bars are SEM, ^∗^*p* < 0.05.

On day 1, immediately following the reading of the text, participants gave an emotional evaluation of the text (6.23 ± 0.21 for control participants, 6.95 ± 0.25 in the teeth group). For this initial emotional evaluation on day 1, no significant effect of condition, sex or interaction was found. We calculated mean scores for day 1 and day 2 and then calculated the difference between these 2 days, representing the evolution of emotion and memory toward the text (day2 scores-day1 scores). We found a significant decrease in negative emotional evaluation in smiling participants as compared to control participants (**Figure 2**). This was confirmed by a three-way ANOVA (group, sex, marital status) that showed a significant group effect [*F*(1,76) = 5.28, *p* = 0.024], but no sex [*F*(1,76) = 2.49, *p* = 0.11], nor marital status effect [*F*(1,76) = 0.74, *p* = 0.39].

Concerning memory scores, all groups showed a slight decrease in their memory scores on day 2. The teeth group had a score of 4.83 (±0.29) on day 1 and 4 (±0.23) on day 2, whereas the control group had a score of 4.98 (±0.25) on day 1 and 4.18 (±0.26) on day 2. The difference between memory scores on day 1 and on day 2 is illustrated in **Figure 2**. A two-way ANOVA (group and sex) on the memory scores difference showed no group [*F*(1,80) = 0.02, *p* = 0.86], no sex effect [*F*(1,80) = 2.32, *p* = 0.13] nor sex^∗^group interaction [*F*(1,80) = 1.64, *p* = 0.2].

## Discussion

Our results show that somatic state manipulation *per se* during memory reactivation interferes with further emotional evaluation of a negative memory. Indeed, the contraction of the zygomaticus muscles (unconscious smiling) during memory reactivation provoked a decrease in negative emotional evaluation toward the memory of a sad text as compared to its evaluation immediately following its reading. This suggests that somatic states play a role not only in the evaluation of a current emotional situation (Damasio, 1996; Niedenthal, 2007; Damasio and Carvalho, 2013), but that they also modify the evaluation of an emotionally laden memory. While the contraction of the zygomaticus muscles positively modulated the emotional evaluation of memories, it had no impact on episodic memories (which slightly decreased for all groups between day 1 and day 2), neither on the general emotional state of the participants (as shown by the PANAS assessments). Moreover, participants with activated zygomaticus muscles evaluated the text as less negative, even though they rated the drawing task as more unpleasant and difficult than the hand control participants. In other words, the zygomaticus activated group’s more positive evaluation of emotionally laden memories is independent from the conscious feeling accompanying the manipulation. These results have several clinical and theoretical implications related to the present results distinguishing between episodic memories and their emotional counterparts.

First, over the past decade, memory malleability has become a topic of great interest for the understanding and treatment of mental disorders (such as post traumatic stress disorder) in which strong negative emotional memories play a crucial role (Pitman et al., 2002; Kindt et al., 2009; Donovan, 2010; Debiec et al., 2011; Lonergan et al., 2012; Schwabe et al., 2012). Though experimental use of propranolol allows for a better understanding of how consolidated memories can still be disrupted or reconsolidate, propranolol acts as a pharmacological amnesic agent. Thus, using it to erase memories that are part of a person’s personality – even if the memories are threatening – raises serious ethical issues (Henry et al., 2007; Donovan, 2010). If somatic states manipulation suffices to modify the emotional component associated with a memory, without affecting its content (episodic memory), our results suggest that ingesting amnesic drugs may not be required to modify the evaluation of negative memories. This is in line with the proposition that memory malleability, through recollection, does not necessarily represent an occasion for inducing amnesia (Gisquet-Verrier and Riccio, 2012). Rather, it opens a window for old negative memories to be associated to new emotional tones.

Second, in the present experiment we found smiling to modulate the affective tone associated with a negative memory. Since one of the main tenets of embodied cognition posits that cognitive functions are influenced by bodily states (Shapiro, 2007; Wilson and Foglia, 2011), we propose that the somatic markers involved in the dynamic recollection process can be defined as embodied memories. It is worth noting that these somatic effects might not be exclusively peripheral and may be encoded by the brain mechanisms that underlie facial feedback effects. For instance, subjects whose upper face expressions are blocked by Botox injections report less fear and emotions when viewing fearful stimuli and show a decreased activation in both the amygdala and the dorsal pons (Davis et al., 2010). These subcortical structures have been hypothesized to play a crucial role in the mapping of bodily states (Damasio, 1994; Damasio and Carvalho, 2013). Thus, in agreement with the somatic marker hypothesis as formulated by Damasio, memory is embodied in the sense that the full recollection of a past event necessitates a close interplay between the reactivation of a centrally encoded episodic memory and the peripherally reactivated somatic states that were originally associated to the memory.

Third, the ethical and embodied aspects of memory is of relevance for the treatment of certain pathologies (Ansermet and Magistretti, 2007). As mentioned above, facial feedback manipulation impacts emotional evaluation even if participants found the manipulation difficult and unpleasant. Thus, as participants evaluate the text more positively, their current feelings are “unconsciously” influenced by zygomaticus contraction. These results may partially explain why certain psychotherapeutic settings are beneficial to patients. A similar hypothesis has been proposed to explain Eye Movement Desensitization and Reprocessing (EMDR) efficacy (Schubert et al., 2011). EMDR has been shown to decrease the physiological markers associated to distress. As such, reactivating traumatic experiences while linking them to these relaxing somatic markers should help patients to deal with their threatening memories in a more acceptable manner. Furthermore, this data may be of relevance to explain why “talking cures” are beneficial to traumatized patients (Bion, 1984; Schottenbauer et al., 2008). The present results imply that recalling traumatizing events does not necessarily consolidate the link between the recalled experiences and the threatening feelings associated to them. Even if the recall of traumatic events can be first felt as unpleasant, it could allow one to re-associate threatening memories with more positive emotions (Alberini et al., 2013). Such positive emotions could be “unconsciously” induced by the reassuring psychotherapeutic setting and the psychoanalyst’s “benevolent neutrality.”

Finally, understanding and explaining therapeutic efficacy with regards to somatic marker manipulation still faces several open issues. If some of the psychotherapeutic effects are to be based on a re-association of threatening memories with more pleasant somatic states, then the effect of the manipulation should exhibit a durable effect. In other words, this new association would have to be reconsolidated. However, in the present study, the time between the reactivation and the memory test was approximately 2 min. Though several studies have used similarly short times between the reactivation and assessment for memory reconsolidation (Chan et al., 2009; Chan and LaPaglia, 2013), a 24-h delay is generally considered to be necessary for full memory reconsolidation (Schiller et al., 2010; Schwabe et al., 2012). Further studies that respect the 24-h period should thus be conducted to test whether the new emotional tone has been durably associated to an old memory, i.e., that the new association has been reconsolidated.

## Conflict of Interest Statement

The authors declare that the research was conducted in the absence of any commercial or financial relationships that could be construed as a potential conflict of interest.
